# Impact of Full-Spectrum and Infrared Lighting on Growth, Oxidative Stress, and Cecal Microbiota in Broilers

**DOI:** 10.3390/antiox13121442

**Published:** 2024-11-23

**Authors:** Khawar Hayat, Rongjin Zheng, Li Zeng, Zunzhong Ye, Jinming Pan

**Affiliations:** 1 College of Biosystems Engineering and Food Science, Zhejiang University, Hangzhou 310058, China; 0621216@zju.edu.cn (K.H.);; 2 Ministry of Agriculture and Rural Affairs, Key Laboratory of Intelligent Equipment and Robotics for Agriculture of Zhejiang Province, Hangzhou 310058, China

**Keywords:** broilers, full-spectrum light, infrared light, growth performance, oxidative stress, cecal microbiota

## Abstract

Lighting is crucial for the development of broilers as it affects their growth performance, oxidative stress, and overall health. This study investigates the impact of full-spectrum light, infrared light, and LED white light exposure on the growth performance, oxidative stress markers, and cecal microbiota of medium-growth yellow-feathered broilers. A total of 216 medium-growth yellow-feathered chicks (Yuhuang No. 5), five days old, were randomly divided into three groups: 72 chicks in each group, with three replicates of 24 chicks. The birds were raised under different lighting conditions, including LED infrared light (II), full-spectrum therapy light (FB), and LED white light (CG) until day 87. This experiment comprised the early growth phase and measured critical hormones such as Melatonin (Mel), Growth Hormone (GH), and Growth Hormone Releasing Hormone (GHRH), as well as Malondialdehyde (MDA), Superoxide Dismutase (SOD), and Catalase (CAT). Additionally, this study examined the differences in microbiota diversity and composition. The results demonstrated that LED infrared and full-spectrum light exposure significantly (*p *< 0.05) increased broiler body weight. Particularly, full-spectrum light was effective in comb redness and reducing final comb length and oxidative stress. Furthermore, full-spectrum light improved microbial prosperity and diversity compared with the other lighting conditions. Overall, the findings suggest that full-spectrum lighting is more beneficial for broiler growth, reducing oxidative stress, and promoting gut health compared with LED infrared lighting. These insights can be applied to optimizing broiler farming practices, thereby improving productivity and animal welfare.

## 1. Introduction

Lighting plays a crucial role in broiler development, impacting growth performance, oxidative stress, and overall health. Natural and artificial lighting are environmental factors that exert physiological effects such as vision, regulating reproductive hormones, and affecting social behavior in broilers [1,2]. Through behavioral, physiological, and neurological mechanisms, light contributes significantly to the production and welfare of broilers [3]. Four distinct variables of light (intensity, photoperiod, source, and wavelength) significantly impact poultry by regulating their physiological functions, growth, maturation, and reproductive activities [4]. Multiple studies have investigated how lighting wavelengths and intensities affect poultry production. Different monochromatic light wavelengths and intensities improved poultry parameters [5,6,7,8,9,10]. LED green light incubation improved chicken welfare by reducing pecking behavior in poultry [5]. Further, green light exposure enhanced antioxidant balance in broilers, lowering oxidative stress and boosting feed efficiency [6]. Blue and green monochromatic light boosted testosterone and myofiber proliferation in broilers, increasing body weight and muscle mass [7]. Moreover, blue and red illumination improved layer hen behavior, plumage scores, foot condition, and growth performance, reducing aggression and improving well-being [8,9]. Infrared radiation has been shown to promote numerous health benefits in poultry production. IR beak trimming and spectroscopy improved growth performance, animal welfare, and feed quality [10]. Lighting also helped chickens manage oxidative stress. The capacity of Salmonella enteritidis strains to survive in harsh circumstances, particularly oxidative stress, altered their virulence and poultry health under different lighting conditions [11]. Heat stress and oxidative stress in chickens caused mitochondrial dysfunction [12]. Heat stress-induced oxidative damage reduced broiler and laying hen productivity, particularly liver function [13]. Heat-induced oxidative stress in poultry negatively affected avian liver lipid metabolism and growth and performance [14]. The gut microbiota are another important parameter to consider when assessing performance. It was found that blue and green illumination increased beneficial bacteria and improved small intestine growth [15]. Red and blue monochromatic light combinations boosted cecal T lymphocyte proliferation, improving immunological function and gut microbiota balance in chicks [16]. Light and temperature manipulation can improve thermotolerance in freshly born chicks [17] and reduce stress [18]. Lighting optimization in broiler houses improved feed conversion ratios and mortality rates [19]. Researchers observed that chickens raised under gradual light spectrum variation exhibited better feed conversion ratios and weight gain compared with those under continuous illumination [20]. Furthermore, full-spectrum illumination improved broilers’ growth performance, carcass production, meat quality, and blood components [21]. While the poultry industry commonly uses standard white LED lighting, there is a need to explore lighting sources and regimens that are more conducive to broiler growth. The impact of lighting on broiler growth, oxidative stress, and cecal microbiota is a crucial aspect of poultry production. It is essential to consider the potential long-term consequences of lighting on oxidative stress, metabolic changes, and gut microbiota in poultry production. By investigating the influence of full-spectrum and infrared lighting on broilers throughout their lifecycle, we can gain valuable insights into optimizing growth, reducing stress, and promoting overall health in poultry farming. However, most research has focused on light intensity and photoperiod, with little exploration of full-spectrum and infrared lighting. While infrared studies typically use rats, which differ from chickens, red light has been shown to enhance muscle development in broilers, suggesting that longer-wavelength infrared radiation may also be beneficial. Implementing modern lighting technologies in chicken farming, including LED infrared and full-spectrum treatment, has the potential to significantly improve broiler development, diminish oxidative stress, and enhance gut health, far beyond the efficacy of conventional white LED lighting methods. This groundbreaking approach aims to transform poultry welfare and production by precisely altering light conditions. Evidence from previous studies indicates that different light spectra influence chicken health, growth performance, and oxidative stress via hormonal modulation and microbial community structure. We hypothesise that varying lighting conditions significantly influence the growth performance, oxidative stress levels, comb development, and cecal microbiota composition of medium-speed yellow-feathered broilers, with full-spectrum and infrared lighting offering greater advantages compared with standard LED white light. This study was conducted to investigate the effects of different lighting conditions (LED infrared, full-spectrum therapy, and LED white light) on the performance of medium-speed yellow-feathered broilers. This study investigated stress-related physiological hormones and IGF-1 levels to understand how these lighting conditions influence oxidative stress and its impact on growth performance and comb development. Additionally, we used 16S rDNA amplicon sequencing to investigate the composition of the cecal microbiota, determining how various light exposures affect the microbial community and its functional implications for growth performance and oxidative stress in the host.

## 2. Materials and Methods

### 2.1. Experiment Design

A total of 216 one-day-old medium-growth yellow-feathered chicks (Yuhuang No. 5) weighing 50.0 ± 1.0 g were bought from Zhejiang Lihua Livestock Co., Ltd., Jiaxing City, China. The chicks were randomly allocated to three groups, each containing 72 chicks with three replicates each having 24 chicks, all reared until day 87, and the groups were as follows: birds reared under LED infrared light (IR), birds reared under full-spectrum therapy light (FS), and birds reared under LED white light (WL). During the brooding period, the area designated for each group was 2.25 m^2^ (1.5 × 1.5 m^2^), expanding to 7.14 m^2^ (2.1 × 3.4 m^2^) post-brooding. The areas were segregated using opaque black tarps to prevent light penetration.

### 2.2. Feeding Regimen and Rearing Environment

From 1 to 14 days of age, the chicks were fed the C101 feed formulated for medium-growth chickens. From 15 to 49 days of age, they were offered W303 feed, and from day 50 to 87 of age, the W304 feed was provided. The rearing method employed was floor rearing on dry rice husk bedding. During the brooding period, the temperature was maintained at 34 ± 1 °C, gradually decreasing to 28 ± 1 °C as the chicks aged.

### 2.3. Lighting Management

For the IR Group, the light source device was an infrared LED (Hangzhou Langtuo Biotechnology Co., Ltd., Hangzhou, China); for the FS Group, the device was a full-spectral animal therapy lamp (Arcadia Reptile, Chichester, UK); and for the WL Group, the light source was a white LED (Jiangmen Bolin Lighting Technology Co., Ltd., Jiangmen City, China). Each light source was placed centrally above the rearing area. The light intensity was precisely measured with a lux meter (Hangzhou Optoelectronic Information Co., Ltd., Hangzhou, China). The height of each light source was adjusted so that the vertical radiation intensity measured directly below the lighting equipment was 0.46 ± 0.01 W/m^2^. The diameter of light source bulbs for the treatments were 8.0 cm and length was 15 cm. The photoperiod for each group was set uniformly at 24 h during brooding and L:D = 16:8 (lighting time from 7:00 to 23:00) after brooding.

### 2.4. Broiler Growth Performance Evaluation

After randomly dividing into groups, the initial body weight of each bird was measured using an electronic balance (BEL Engineering, Monza, Italy) with a precision of 0.1 g, and the final body weight was measured on the final day (day 87). After that, blood from the wing vein was collected for appearance quality inspection. After dissection, the entire breast (without skin and bone), gizzard (without digesta and keratin epithelium), spleen, liver (without gallbladder), and heart were separated and weighed. The axial tibia length of each bird was measured using a digital vernier caliper (Nanjing Suzhace Measurement Instruments Co., Ltd., Nanjing, China) with a precision of 0.1 mm.

### 2.5. Detection of Comb Development

A color space model was used for measuring comb chromaticity using a CR-400 Chroma Colorimeter (Konica Minolta, Inc., Dongguan, China). The L-value, a-value, and b-value of each bird’s comb were obtained. In the lab color space, L represents brightness, where higher values indicate greater brightness; a represents the green-to-red component, changing from negative to positive as color shifts from green to red; and b represents the blue-to-yellow component, changing from negative to positive as color shifts from blue to yellow. The height of the comb (the distance from the tip of the comb to the base) and the length of the comb were measured using a digital vernier caliper.

### 2.6. Blood Sample Collection

Approximately 2 mL of sub-wing vein blood was collected from the birds using a syringe (Jiangxi Hongda Medical Device Group Co., Ltd., Nanchang City, China). Centrifugation at 3000 rpm for 10 min was performed using a centrifuge (Thermo Fisher, Waltham, MA, USA) to separate serum from the blood cells. The supernatant was transferred using a pipette to Eppendorf tubes (Eppendorf, Hamburg, Germany) and stored at −80 °C for later use.

### 2.7. Collection of Broilers Cecal Contents

From each replicate, 3 broiler chickens were humanely euthanized by cervical dislocation. The ceca were then dissected under sterile conditions, and the cecal contents were collected and stored at −80 °C for DNA extraction and PCR amplification.

### 2.8. DNA Extraction

Microbial DNA extraction was performed using the E.Z.N.A.^®^ Stool DNA Kit from Omega Bio-Tek, Norcross, GA, USA, following a detailed procedure. Initially, 200 mg of fecal sample was added to a 2 mL microcentrifuge tube with 200 mg of glass beads and placed on ice. Then, 54 µL of SLX Mlus buffer was added, and the mixture was vortexed until homogenized. Next, 60 µL of DS buffer and 20 µL of proteinase K solution were introduced, followed by vortexing and incubation at 70 °C for 10 min with intermittent vortexing. Afterward, 200 µL of SP2 buffer was added, vortexed for 30 s, and the sample was placed on ice for 5 min to precipitate. Centrifugation at 13,000× *g* for 5 min allowed for the transfer of 400 µL of supernatant to a new tube. Then, 200 µL of cHTR reagent was added, vortexed, and allowed to stand before centrifugation. The supernatant was then transferred to another tube, and 250 µL each of BL buffer and 100% ethanol were added and vortexed. The mixture was loaded onto a HiBind^®^ DNA Mini Column, centrifuged, and the supernatant discarded. The column was washed with VHB buffer and DNA wash buffer, followed by additional centrifugation steps to remove impurities. Finally, the DNA was eluted using 100–200 µL of heated elution buffer, centrifuged, and stored at −20 °C.

### 2.9. PCR Amplification

The real-time PCR was done according to the protocol established by Abitayeva and Abeev [22]. The primer sequences were as follows:

F (CCTACGGGNGGCWGCAG)

R (GGACTACHVGGGTWTCTAAT)

The reaction parameters were as follows: initial denaturation at 95 °C for 2 min, followed by 27 cycles of denaturation at 95 °C for 30 s, annealing at 55 °C for 30 s, and extension at 72 °C for 30 s, with a final extension at 72 °C for 5 min. For the second round of amplification, primers compatible with Illumina’s bridge PCR were introduced. The PCR reaction parameters were as follows: initial denaturation at 95 °C for 3 min, followed by 5 cycles of denaturation at 95 °C for 15 s, annealing at 55 °C for 15 s, and extension at 72 °C for 30 s, with a final extension at 72 °C for 5 min, ending with storage at 10 °C.

### 2.10. Library Construction and Sequencing

Quantitative analysis of the purified PCR products was conducted using the Qubit3.0 DNA Assay Kit (Thermo Fisher, Waltham, MA, USA). The amplicon library was sequenced on the Novaseq 6000 with a paired-end 250 base read strategy. Raw reads were deposited in the NCBI Sequence Read Archive (SRA) database.

### 2.11. Sequence Data Processing

To obtain more accurate and reliable information, raw sequencing data underwent merging and noise filtering. Based on the effective data, we performed Amplicon Sequence Variants (ASVs) clustering and taxonomic classification analyses. Abundance calculations and Alpha diversity analyses were conducted using ASVs, yielding information about species richness and evenness within samples. Furthermore, through dimensionality reduction analyses such as Principal Coordinates Analysis (PCoA), Principal Component Analysis (PCA), and Non-metric Multidimensional Scaling (NMDS), as well as sample cluster tree displays, differences in community structure between different samples or groups were investigated. To further explore community structure differences among grouped samples, statistical methods like Adonis were employed to test for significant differences in species composition and community structure among groups, with *p* < 0.05 indicating statistically significant differences between groups. By correlating ASVs’ annotation results with relevant functional databases, PICRUST2 software was used to perform functional prediction analysis on microbial communities in ecological samples. The pipeline used for sequencing data processing was Amplicon Sequence Variants (ASVs) clustering and taxonomic classification. NCBI Sequence Read Archive (SRA) database was used for classification in this process.

### 2.12. Enzyme-Linked Immunosorbent Assay (ELISA)

Microplate Reader Infinite F50 Tecan Chicken Cortisol ELISA Kit, Nanjing Jiancheng Bioengineering Institute; Chicken Malondialdehyde (MDA) ELISA Kit, Nanjing Jiancheng Bioengineering Institute; Chicken Superoxide Dismutase (SOD) ELISA Kit, Nanjing Jiancheng Bioengineering Institute; Chicken Catalase (CAT) ELISA Kit, Nanjing Jiancheng Bioengineering Institute; Chicken Insulin-like Growth Factor 1 (IGF-1) ELISA Kit, Nanjing Jiancheng Bioengineering Institute; Chicken Melatonin ELISA Kit, Nanjing Jiancheng Bioengineering Institute; Chicken Growth Hormone (GH) ELISA Kit, Nanjing Jiancheng Bioengineering Institute; Chicken Growth Hormone-Releasing Hormone (GHRH) ELISA Kit, Nanjing Jiancheng Bioengineering Institute. In this experiment, to quantify the levels of above-mentioned hormones, ELISA method was used. The process comprised precoating microtiter plates with specified antibodies, then adding 50 μL of standard solutions, 10 μL of test samples, and 40 μL of sample diluent in designated wells. After incubation with 100 μL of enzyme-labeled detection antibodies at 37 °C for 60 min, plates were carefully washed to eliminate unbound materials. Color development began by adding equal volumes of substrates A and B, incubation for 15 min in the dark at 37 °C, and then stopping the reaction with a stop solution. Finally, the optical density (OD) was determined at 450 nm using a microplate reader, with the intensity of color development corresponding to the concentration of the target analyte.

### 2.13. Statistical Analysis

The performance, blood data, and organ and tissue weights were analyzed using a one-way ANOVA through SPSS 23.0 statistical software. In cases where ANOVA revealed significant differences, the Tukey HSD test was used to determine difference between treatment means. All the results are presented as mean (±) standard error of the mean (SEM). A *p*-value less than 0.05 indicated statistically significant differences among groups. All bar charts for the MDA levels analysis, SOD and CAT levels, and activity analysis and intergroup differences in IGF-1 levels and intergroup differences in GH and GHRH levels were generated using the GraphPad Prism v.7.

## 3. Results

### 3.1. Growth Performance

As shown in Table 1, the final body weight of broilers in group IR and the FS group was significantly (*p* < 0.05) higher than that in the WL group. There was no significant difference between the IR and FB groups. There were no significant differences in the final tibia length, heart weight, liver weight, spleen weight, or organ indices (percentage of organ weight relative to total body weight) except for the gizzard among the groups in Table 2. There were no significant inter-group differences in breast muscle weight among the different lighting treatments (Table 2). The gizzard weight of the FS group was significantly (*p* < 0.05) higher than the IR and WL groups, while there was no statistical difference between the IR and WL groups Table 2.

### 3.2. Oxidative Stress Parameters

The detail results of oxidative stress parameters are given below. 

### 3.3. MDA Levels Analysis

**Figure 1:**
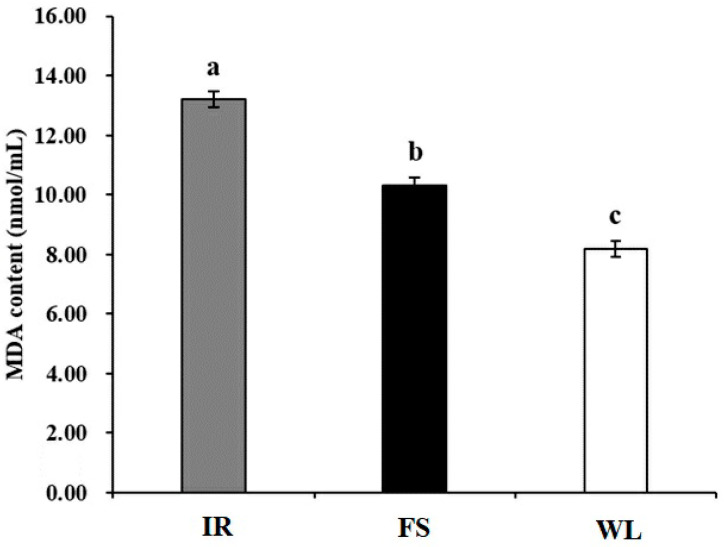
Blood MDA level of broilers in different light treatment groups. IR: birds reared under LED infrared light; FS; birds reared under full-spectrum therapy light; WL; birds reared under LED white light. Superscripts a, b, and c indicate statistical differences. A *p* < 0.05 is considered a statistically significant difference.

The MDA levels in the IR group were significantly higher than those in the FS and WL groups, and the FS group MDA levels were significantly (*p* < 0.05) higher than those in the WL group (Figure 1).

### 3.4. SOD and CAT Levels and Activity Analysis

**Figure 2:**
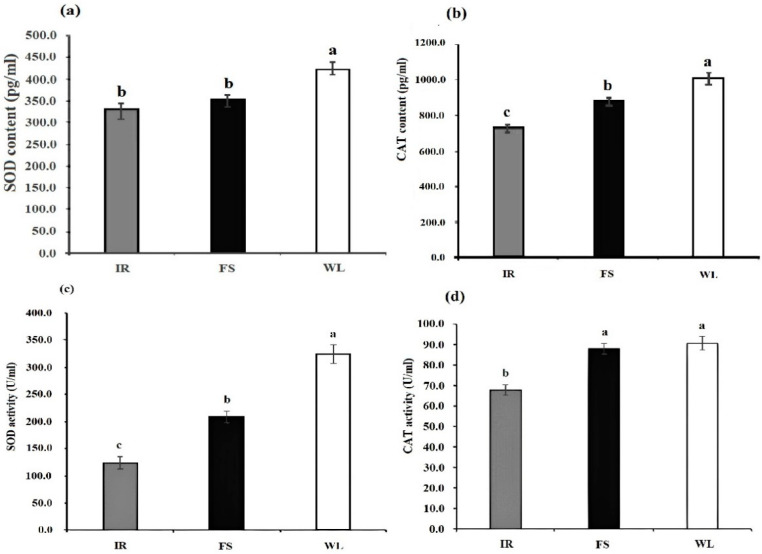
Levels and activities of antioxidant enzymes in the blood of broilers under different light treatments: (**a**) SOD level; (**b**) CAT level; (**c**) SOD activity; (**d**) CAT activity. IR: birds reared under LED infrared light; FS; birds reared under full-spectrum therapy light; WL; birds reared under LED white light. Superscripts a, b, and c indicate statistical differences. A *p* < 0.05 is considered a statistically significant difference.

As illustrated in Figure 2, the WL group exhibited significantly higher SOD levels than the IR and FS groups. There was no significant difference between IR and FS groups for SOD levels. The CAT levels were significantly higher in the WL group compared with the FS group, and the FS group had significantly higher CAT levels than the IR group (*p* < 0.05). So, both the WL and FS groups had significantly higher CAT activity than the IR group, but there was no significant difference between the WL and FS groups (*p* < 0.05).

### 3.5. Analysis of Intergroup Differences in IGF-1 Levels

**Figure 3:**
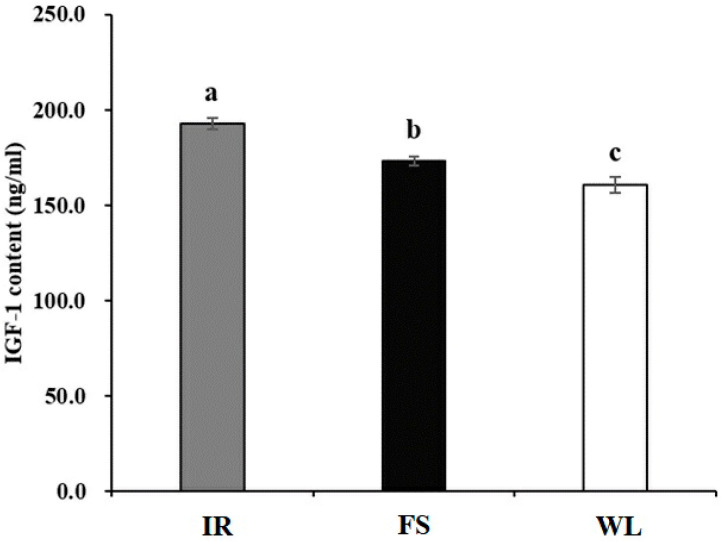
Blood IGF-1 level of broilers in different light treatment groups. IR: birds reared under LED infrared light; FS; birds reared under full-spectrum therapy light; WL; birds reared under LED white light. Superscripts a, b, and c indicate statistical differences. A *p* < 0.05 is considered a statistically significant difference.

As illustrated in Figure 3, the IR group exhibited significantly higher IGF-1 levels compared with both the FS and WL groups. Furthermore, both group IR and FS showed significantly higher levels of IGF-1 than group WL (*p* < 0.05).

### 3.6. Analysis of Intergroup Differences in Melatonin (Mel) Levels

**Figure 4:**
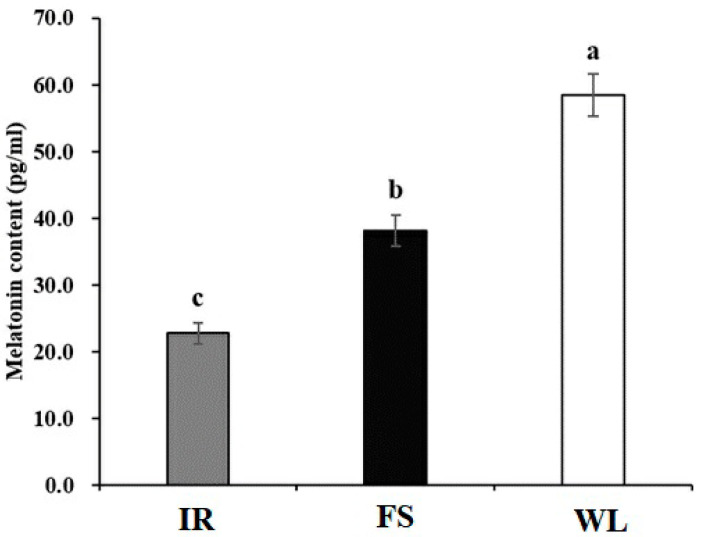
Blood Melatonin level of broilers in different light treatment groups. IR: birds reared under LED infrared light; FS; birds reared under full-spectrum therapy light; WL; birds reared under LED white light. Superscripts a, b, and c indicate statistical differences. A *p* < 0.05 is considered a statistically significant difference.

As illustrated in Figure 4, group IR showed significantly lower Melatonin levels in broiler chicken blood than group FS. Moreover, both IR and FS groups showed significantly lower levels than Group CG (*p* < 0.05).

### 3.7. Analysis of Intergroup Differences in GH and GHRH Levels

**Figure 5:**
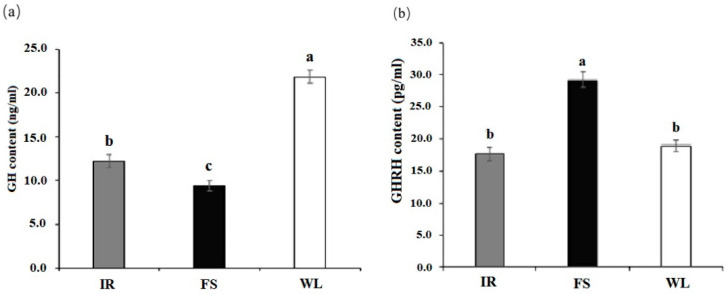
Blood GH level and GHRH level of broilers in different light treatment groups: (**a**) GH level; (**b**) GHRH level. IR: birds reared under LED infrared light; FS; birds reared under full-spectrum therapy light; WL; birds reared under LED white light. Superscripts a, b, and c indicate statistical differences. A *p* < 0.05 is considered a statistically significant difference.

As illustrated in Figure 5, the GH levels in group WL were significantly higher than those in group IR and FS, and the FS group showed significantly higher levels than group IR. Regarding GHRH, group FS had significantly higher levels than both group IR and WL, with there being no statistical difference between groups IR and WL (*p* < 0.05).

### 3.8. Comb Development

As presented in Table 3, there were significant intergroup differences in the redness, length, and height of combs under different lighting treatments. In terms of the L (brightness) component, no significant differences existed among the groups, with group IR having the highest brightness of combs and group FS the lowest. For the redness component, group FS showed significantly higher values than group WL. In terms of comb length, group IR had a significantly longer comb than group FS. For comb height, group IR had significantly greater heights than those in group FS, with no significant differences with group WL.

### 3.9. Impact of Different Environmental Lightings on Cecal Microbiota Composition

This study evaluated the effects of different lighting environments on the cecal microbiota composition of moderately growing yellow-feathered broiler chickens. By comparing the rarefaction curves among the three groups, including the observed features index, chao1 index, and Shannon index, it was found that with the increase in sequencing depth, the curves tended to level off, indicating that the sequencing depth had largely covered all species within the samples (Supplementary Figure S1). In addition, alpha diversity analysis was employed to determine species richness and microbial community diversity. There were no significant differences in various alpha diversity indices among the three light treatment groups (*p* > 0.05) (Supplementary Figure S2). Beta diversity measurements, including Principal Component Analysis (PCA), Principal Coordinates Analysis (PCoA), and Non-metric Multidimensional Scaling (NMDS), were used to ascertain the dispersion of microbes across different light treatment groups (Supplementary Figure S3). If two samples are closely positioned, a similar composition of species is indicated between them. PCA and PCoA analyses based on weighted UniFrac distances revealed the segregation of samples among the three groups. According to PERMANOVA (Permutational Multivariate Analysis of Variance), the microbial community profiles significantly differed among the three groups (AdonisR^2^ = 0.172, *p* = 0.005).

#### 3.9.1. Influence of Different Environmental Lightings on Phylum-Level Abundance in Cecal Microbiota

##### Inter-Group Abundance Differences at the Phylum Level in Cecal Bacteria

**Figure 6:**
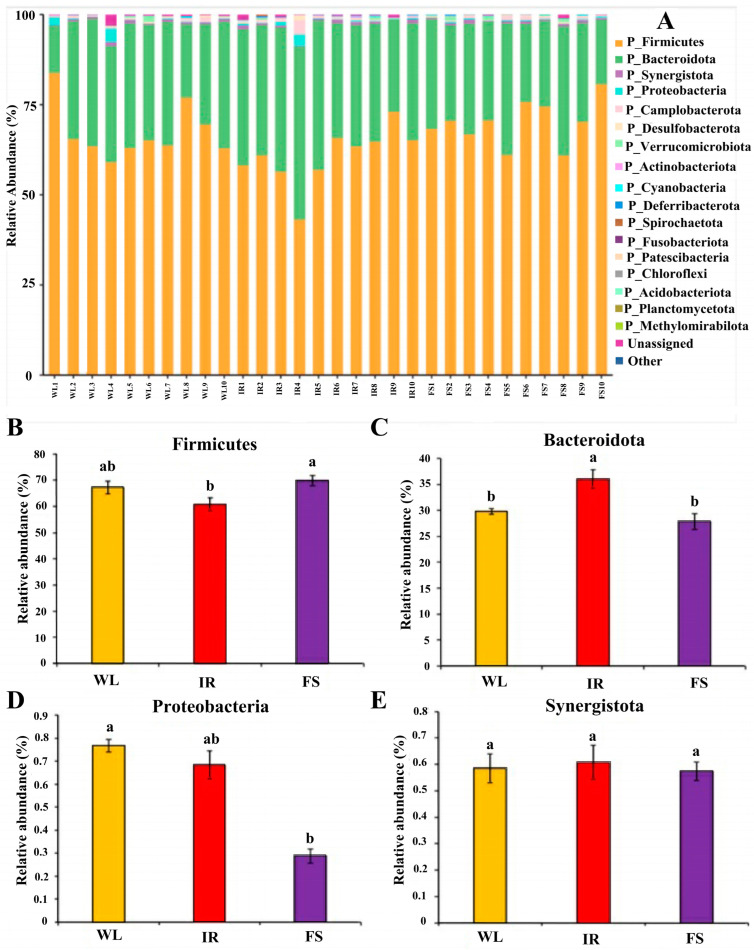
Relative abundance of cecal microbiota at the phylum level. The first four main phyla: (**A**) Relative abundance of cecal microorganisms at the phylum level in all samples; (**B**) Firmicutes; (**C**) Bacteroidota; (**D**) Synergistota; (**E**) Proteobacteria; IR: infrared group FS: Full-spectrum group; WL: white light group (control group). Superscripts a, b indicate statistical differences. A *p* < 0.05 is considered a statistically significant difference.

As illustrated in Figure 6, the dominant bacteria in the ceca of broilers under different lighting conditions were Firmicutes (WL: 67.27%, IR: 60.73%, FS: 69.89%) and Bacteroidetes (WL: 29.77%, IR: 35.98%, FS: 27.86%). Notably, the relative abundance of Firmicutes in the FS group was significantly higher than in the IR group (*p* < 0.05). The relative abundance of Bacteroidetes in the IR group was significantly higher than in the WL and FS groups (*p* < 0.05), while no significant difference was observed between the WL and FS groups. There were non-significant differences in the abundances of Synergistetes and Proteobacteria among the groups.

#### 3.9.2. Intergroup Differences of Cecal Microbiota in Broilers

**Figure 7:**
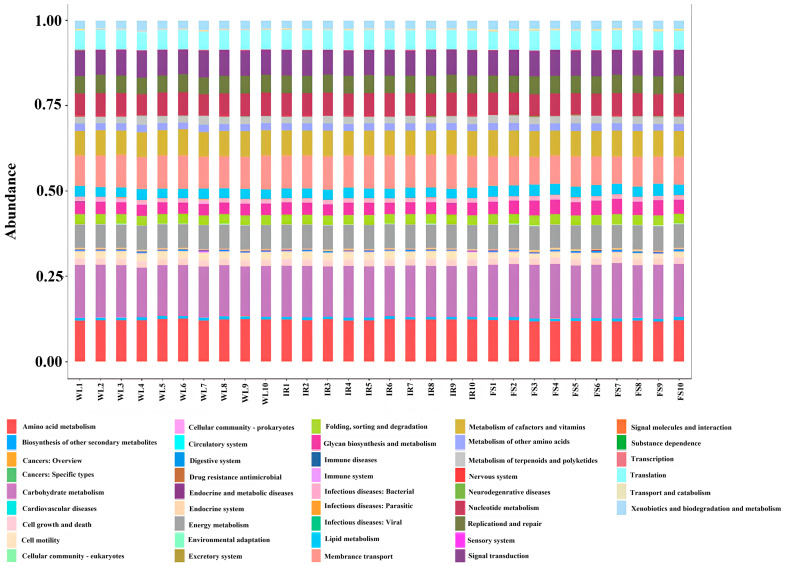
Tax4Fun secondary path annotation results IR: infrared radiation group; FS: full-spectrum radiation group; WL: white light group (control group).

**Figure 8:**
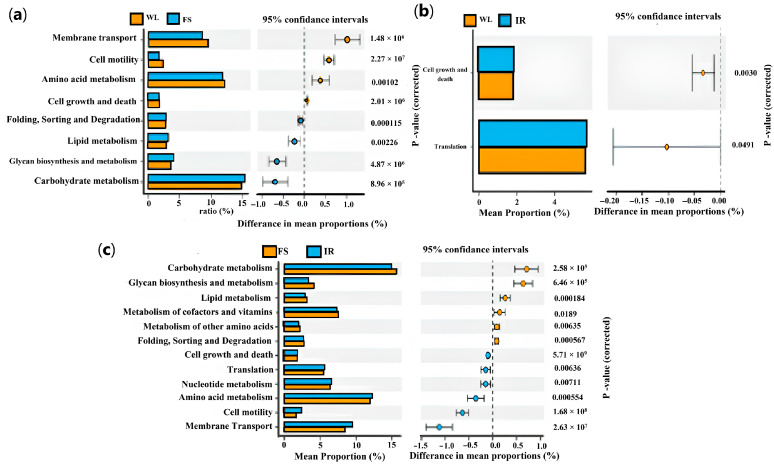
KEGG Abundance Data of the group under full-term illumination: (**a**) WL and FS; (**b**) WL and IR; (**c**) IR and FS. IR: infrared radiation group; FS: full-spectrum radiation group; WL: white light group (control group).

Under the continuous light exposure regimen, the functional profiles of individual samples were remarkably similar (Figure 7). Figure 8 indicates that, compared with the WL, the FS group had significantly upregulated functions related to folding, sorting, degradation, lipid metabolism, the metabolism of polysaccharides, and carbohydrate metabolism (*p* < 0.01), while membrane transport, cell motility, amino acid metabolism, and cellular growth and death functions were significantly downregulated (*p* < 0.01). Compared with the WL group, the IR group showed a significant upregulation in cellular growth and death functions (*p* < 0.01) and translational functions (*p* < 0.05). The FS group compared with the IR group had significantly upregulated carbohydrate metabolism (*p* < 0.01), synthesis and metabolism of polysaccharides (*p* < 0.01), lipid metabolism (*p* < 0.01), co-factor and vitamin metabolism (*p* < 0.05), folding, sorting, and degradation functions (*p* < 0.01). On the other hand, cellular growth and death functions, translational functions, nucleotide metabolism, amino acid metabolism, cell motility, and membrane transport functions were significantly downregulated (*p* < 0.01).

## 4. Discussion

In the present study, medium-speed yellow-feathered broiler chicks were selected as experimental subjects to investigate the effects of full-term lighting on growth and development, oxidative stress, and microbiota properties. Considering that demand for growth changes significantly with age, mechanisms regulating GH patterns undergo feedback adjustments throughout the animal’s entire life span [23]. Therefore, it is reasonable that environmental stimuli provided by lighting treatments have varying impacts on the growth and development of broiler chickens. Under full-term lighting conditions, the body weights of broiler chickens in group IR and group FS were significantly higher than those in WL group. This indicates that full-term lighting has a better growth-promoting effect on mid-speed yellow-feathered broilers. Broiler chickens in the IR group had significantly lower levels of blood antioxidant enzymes (SOD and CAT) than those in group FS, and their MDA was significantly higher than in groups FS and WL. This indicates that full-term LED infrared lighting deepens oxidative stress in broiler chickens, and full-spectrum therapeutic lamp irradiation did not improve the mitigation of oxidative stress. This suggests that, in terms of reactive oxygen species and alleviating oxidative damage, full-term full-spectrum therapeutic lamp lighting neither showed better results nor deteriorated. LED infrared lighting did not allow the broiler chickens to adapt to that lighting environment. Additionally, the effects of lighting systems on oxidative stress and immunity in the perspective of Mel indicated that LED infrared lighting affected the secretion of Mel in broiler chickens, potentially hurting their antioxidant function systems. However, full-spectrum therapeutic lamp lighting could elevate Mel levels in broiler chicken blood, thereby partially restoring their antioxidant capacity and immune function. This study also measured the levels of GH and GHRH in broiler chicken blood, finding no simple linear relationship with IGF-1 levels. This suggests that GH indirectly promotes growth and development through IGF-1, and complex mutual regulation and feedback mechanisms exist among these growth-related hormones. The intricate gut microbiome is crucial for its host, impacting the digestion of feed [24], limiting pathogen growth or adhesion to the intestinal surface [25], and producing metabolites that the host cannot synthesize. Generally, higher microbial diversity is associated with healthier host states, as reduced diversity in the bacterial community structure appears to weaken its resilience against perturbations [26]. Decreased microbial diversity has been linked to various gut disease states [27], and the disruption of the gut microbiome structure leading to the elimination of beneficial bacterial subgroups often results in the overgrowth of pathogens and a significant loss of microbial diversity [28]. Numerous reports indicate that microbes exhibit strong responses to light [29], such as changes in gut microbiome composition due to light pollution caused by artificial nighttime lighting [30]. Notable differences were seen in microbial richness and diversity, as the FS and IR groups exhibited substantial enhancements in species richness and diversity within the cecal microbiota of broilers. Moreover, based on beta diversity analyses, the microbial community compositions displayed even more pronounced differences among the groups. This suggests that continuous lighting regimens, beginning from the chick stage with either LED infrared or full-spectrum therapeutic light treatments, may be more conducive to enhancing microbial richness and diversity in the ceca of broilers, thereby promoting a healthier state for the host. Phylum-level species relative abundance analyses further indicate that different lighting treatments and varying durations of light exposure affect the composition of the cecal microbiota. Among the groups, the predominant phyla were Firmicutes and Bacteroidetes. Firmicutes, mostly Gram-positive bacteria, many of which produce butyrate, which is considered a direct energy source for colonocytes [31] and has anti-inflammatory effects due to its inhibition of Nuclear Factor Kappa-B (NF-κB) activity [32]. Butyrate also enhances colonic defense barriers by increasing mucin production and the generation of host antimicrobial peptides. Additionally, butyrate improves body weight in broilers and inhibits the growth of Salmonella and Clostridium perfringens [33]. Bacteroidetes, being Gram-negative bacteria, are involved in numerous gut metabolic activities. Due to their primary energy source being polysaccharides, an increase in their abundance may contribute to enhanced glycolysis in broilers [16], although some members may exacerbate host inflammation. It has been suggested that the ratio of Firmicutes to Bacteroidetes (F/B ratio) is a critical indicator of gut microbiome health [34], and studies showed that the F/B ratio is significantly correlated with the production of short-chain fatty acids and increased gut energy [35]. A higher F/B ratio may reflect a healthier gut metabolic state and stronger antioxidant capacity. Under the rearing period lighting regime, the FS group had a significantly higher relative abundance of Firmicutes than the IR group and a slightly higher abundance than the WL group. Additionally, the FS and WL groups had significantly lower abundances of Bacteroidetes than the LED infrared group, indicating a higher F/B ratio in the FS group. This suggests that the FS group’s broilers had superior gut health status and antioxidant levels compared with those in the IR group. This could be attributed to the differential effects of lighting treatments on the composition and relative abundance of the cecal microbiota, thus altering gut function and metabolic capabilities, which in turn affects the host’s antioxidant capacity. Host metabolism, diet, and health reciprocally influence the cecal microbiota, maintaining a feedback regulatory relationship. Under the continuous lighting regime, the relative abundances of Firmicutes decreased in all groups, whereas those of Bacteroidetes increased to varying degrees. This implies that continuous light coverage throughout the rearing period reduces the F/B ratio in the ceca of broilers, altering gut function and antioxidant levels.

Indeed, studies propose that the core differences among individuals’ gut microbiomes are not determined by the types of bacteria present but rather by the functional variations they exhibit [36]. KEGG pathway analysis revealed that there were differences in gene enrichments between the FS and IR groups: the FS group had higher abundances of genes involved in metabolism, including amino acid synthesis and carbohydrate metabolism. Similarly, under the continuous lighting regime, the FS group had significantly higher abundances of genes involved in polysaccharide metabolism and carbohydrate metabolism compared with the WL and IR groups. Amino acid synthesis has been shown to aid muscle development, and carbohydrate metabolism plays a crucial role in maintaining antioxidant levels. This suggests that full-spectrum light helps to enhance carbohydrate metabolism and amino acid synthesis functions in the cecal microbiota of broilers, thereby improving their antioxidant levels. This implies that full-spectrum light promotes growth and development in broilers while also supporting the maintenance of antioxidant function and alleviation of oxidative stress.

## 5. Conclusions

The results of this study on the effects of various lighting conditions, LED infrared, full-spectrum therapy, and standard LED white light, on broilers showed that tailored lighting can greatly promote growth, reduce oxidative stress, and improve gut health. Broilers exposed to infrared and full-spectrum lighting had greater final body weights, and full-spectrum lighting also decreased oxidative stress and enhanced microbial diversity, showing the potential to improve overall health and productivity. Different lighting conditions alter hormonal levels further underscoring the importance of light in hormonal regulation, which could further impact growth and stress responses. The present results can apply practically in the poultry industry, suggesting that an improved and optimized lighting environment can lead to better growth outcomes and animal welfare. To improve lighting programs and poultry management practices, future studies should concentrate on long-term effects on meat quality and the ideal conditions for poultry breeds.

## Figures and Tables

**Table 1:** Body weight changes of broilers in different light treatment groups.

		Group		
Item	IR Group	FS Group	WL Group	*p* Value
Initial body weight (g)	50.0 ± 1.0 g	50.0 ± 1.0 g	50.0 ± 1.0 g	
Pre-slaughter weight (g)	2627.4 ± 56.8 ^a^	2718.1 ± 75.6 ^a^	2355.5 ± 64.5 ^b^	0.037

IR Group: birds reared under LED infrared light; FS; birds reared under full-spectrum therapy light; WL; birds reared under LED white light. Superscripts a, b indicate statistical differences. A *p* < 0.05 is considered a statistically significant difference.

**Table 2:** Weight of organs and tissues and organ index.

		Group		
Organ	IR	FS	WL	*p* Value
Axial tibial length (mm)	105.2 ± 2.7	105.1 ± 2.4	101.6 ± 2.2	*p* > 0.05
Heart weight (g)	16.9 ± 2.6	17.4 ± 1.7	15.6 ± 0.6	*p* > 0.05
Liver weight (g)	77.8 ± 8.0	76.8 ± 5.9	73.1 ± 7.1	
Spleen weight (g)	4.7 ± 0.4	6.0 ± 0.7	4.4 ± 0.5	*p* > 0.05
Gizzard weight (g)	57.9 ± 3.6 ^b^	72.5 ± 5.1 ^a^	51.0 ± 2.8 ^b^	0.003
Chicken breast weight (g)	179.7 ± 12.6	185.3 ± 6.8	161.3 ± 7.7	*p* > 0.05
Heart index (%)	0.64 ± 0.10	0.64 ± 0.06	0.66 ± 0.03	*p* > 0.05
Liver index (%)	2.96 ± 0.30	2.83 ± 0.22	3.10 ± 0.30	*p* > 0.05
Spleen index (%)	0.18 ± 0.02	0.22 ± 0.03	0.19 ± 0.02	*p* > 0.05
Musculo gastric index (%)	2.20 ± 0.14	2.67 ± 0.19	2.17 ± 0.12	*p* > 0.05
Breast index (%)	6.84 ± 0.48	6.82 ± 0.25	6.85 ± 0.33	*p* > 0.05

IR Group: birds reared under LED infrared light; FS; birds reared under full-spectrum therapy light; WL; birds reared under LED white light. Superscripts a, b indicate statistical differences. A *p* < 0.05 is considered a statistically significant difference.

**Table 3:** Color, length, and height of broiler comb in different light treatment groups.

		Group		
Item	IR	FS	WL	*p* Value
The final chromaticity L value	41.4 ± 0.8	40.7 ± 0.7	41.3 ± 0.7	*p* > 0.05
Final chroma a-value	32.5 ± 1.0 ^ab^	34.7 ± 0.6 ^a^	31.8 ± 0.8 ^b^	*p* = 0.043
Final chroma b-value	22.4 ± 0.7	22.3 ± 0.4	22.3 ± 0.6	*p* > 0.05
Final comb length (mm)	106.3 ± 4.0 ^a^	95.4 ± 2.7 ^b^	103.3 ± 3.5 ^ab^	*p* = 0.036
Final comb height (mm)	58.7 ± 7.9 ^a^	48.9 ± 1.9 ^b^	55.5 ± 3.0 ^ab^	*p* = 0.046

IR: birds reared under LED infrared light; FS; birds reared under full-spectrum therapy light; WL; birds reared under LED white light. Superscripts a, b indicate statistical differences. A *p* < 0.05 is considered a statistically significant difference.

## Data Availability

The datasets used and/or analyzed during the current study are available from the corresponding author on reasonable request.

## Supplementary Materials

The following supporting information can be downloaded at: https://www.mdpi.com/article/10.3390/antiox13121442/s1, Supplementary Figure S1: Rarefaction and rank abundance curve of the group during the rearing period: observed features index; (b) chao1 index; (c) Shannon index; (d) rank abundance curve; IR: infrared radiation group; FS: full-spectrum radiation group; WL: white light group (control group). Supplementary Figure S2: Alpha diversity index of different groups: (a) ace index; (b) hao1 index; (c) Shannon index; (d) Simpson index. Outliers are marked with “o”. IR: infrared radiation group; FS: full-spectrum radiation group; WL: white light group (control group). Supplementary Figure S3: Beta diversity analysis of the groups: (a) PCA; (b) PCoA; NMDS analysis. IR: infrared radiation group; FS: full-spectrum radiation group; WL: white light group (control group).
