# Supplementary material for: Impact of Full-Spectrum and Infrared Lighting on Growth, Oxidative Stress, and Cecal Microbiota in Broilers
Source: Antioxidants (Basel). 2024 Nov 23;13(12):1442. doi: 10.3390/antiox13121442 (PMC11672630; doi:10.3390/antiox13121442)
Supplement: Supplementary file 1 [file antioxidants-13-01442-s001.zip › antioxidants-3279215-supplementary.pdf]

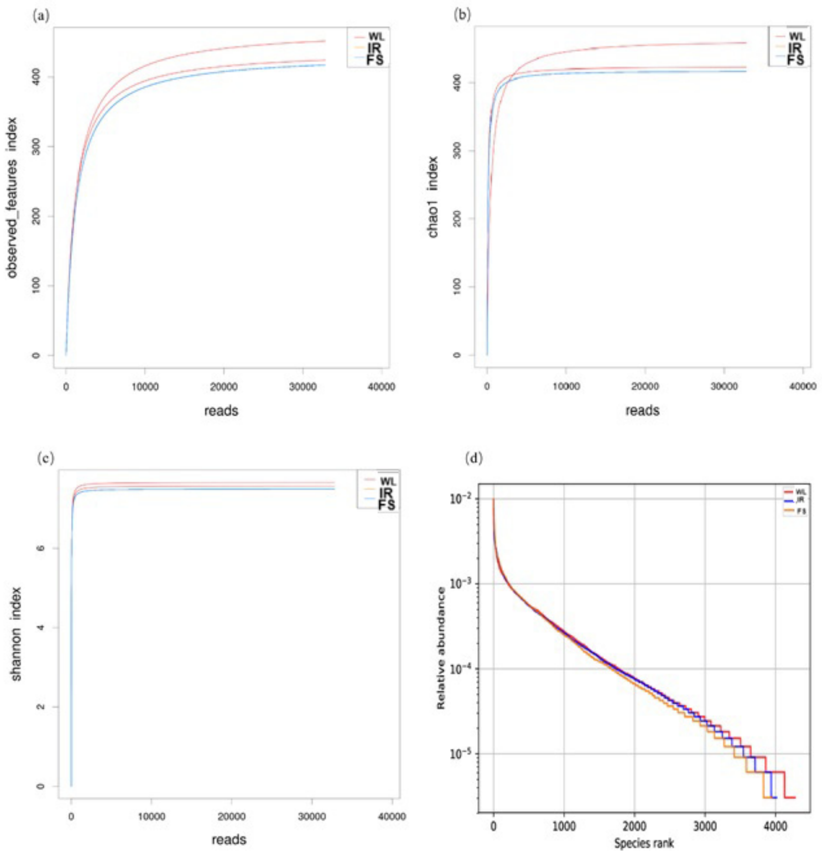

Supplementary Figure S1. Rarefaction and rank abundance curve of the group during the rearing period: observed features index; (b) chao1 index; (c) shannon index; (d) rank abundance curve; IR: infrared radiation group; FS: full spectrum radiation group; WL: white light group (control group).

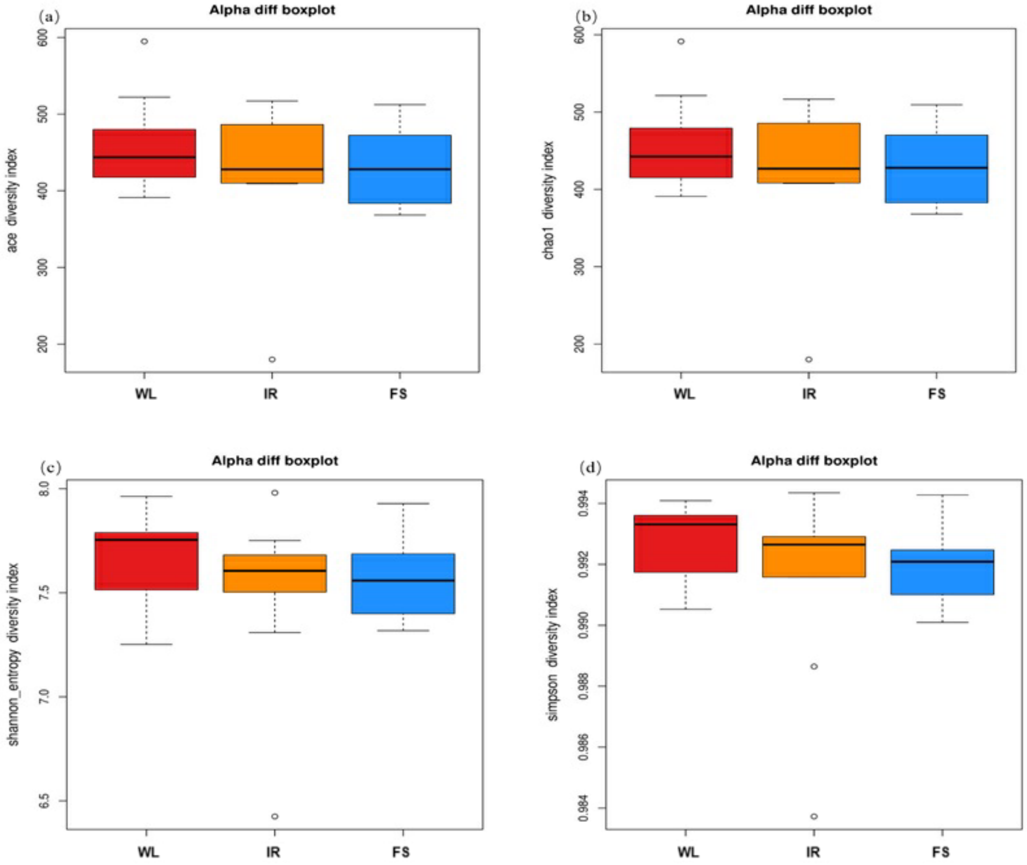

Supplementary Figure S2. Alpha diversity index of different groups: (a) ace index; (b) hao1 index; (c) shannon index; (d) simpson index. Outliers are marked with "o". IR: infrared radiation group; FS: full spectrum radiation group; WL: white light group (control group).

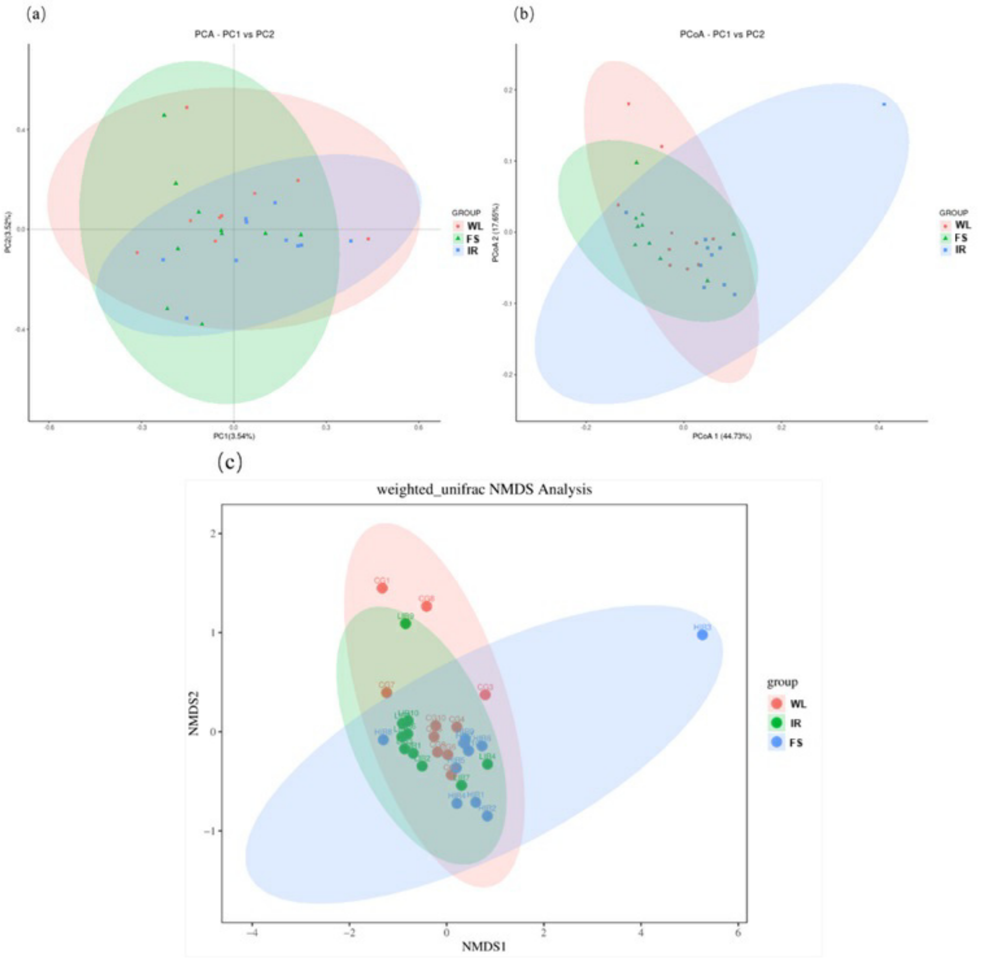

Supplementary Figure S3. Beta diversity analysis of the groups: (a) PCA; (b) PCoA; NMDS analysis. IR: infrared radiation group; FS: full spectrum radiation group; WL: white light group (control group)
